# Evaluating Patient-Reported Outcome Measurement in Fibromyalgia Clinical Trials: A Meta-Analysis of Alignment with IMMPACT Recommendations

**DOI:** 10.21203/rs.3.rs-10425582/v1

**Published:** 2026-07-29

**Authors:** Micaylon Moore, Hayden Keeler, Lauren Kocour, Noah Calvert, Makalie Lackey, Tanner Livsey, Ryan Langerman, Amy Nguyen, Arnab Mukherjee, Alicia Ito Ford, Matt Vassar

**Affiliations:** Oklahoma State University Center for Health Sciences; Oklahoma State University Center for Health Sciences; Oklahoma State University Center for Health Sciences; Oklahoma State University Center for Health Sciences; Oklahoma State University Center for Health Sciences; Oklahoma State University Center for Health Sciences; Oklahoma State University Center for Health Sciences; Oklahoma State University Center for Health Sciences; Oklahoma State University Center for Health Sciences; Oklahoma State University Center for Health Sciences; Oklahoma State University Center for Health Sciences

**Keywords:** Fibromyalgia, Patient-Reported Outcomes, Chronic Pain, Outcome Measurement, Clinical Trial Registries, IMMPACT

## Abstract

**Purpose:**

Fibromyalgia diagnosis and treatment response depend mostly on patient report, making patient-reported outcomes central to trial evidence. The Initiative on Methods, Measurement, and Pain Assessment in Clinical Trials provides recommendations for standardized outcome domains and endorsed patient-reported instruments in chronic pain trials. Whether fibromyalgia trials have converged on a common set of instruments remains unclear. We evaluated instrument standardization and IMMPACT alignment among registered fibromyalgia trials.

**Methods:**

We conducted a cross-sectional meta-research analysis of 916 interventional fibromyalgia trials registered on ClinicalTrials.gov. Outcomes were classified against an inductively built dictionary of patient-reported outcome instruments, with alignment defined as registration of at least one consensus-body-endorsed instrument. Completeness and alignment were assessed relative to the consensus body’s 2005 publication, and cohort assembly and instrument detection were independently verified.

**Results:**

Of 916 eligible trials, 778 (84.9%) named a patient-reported outcome instrument, rising from 47.4% in 2005 to a peak near 98% in 2021. One hundred and two distinct instruments were registered, but only 49.1% of these trials included one the consensus body endorses. The most common measure was the Fibromyalgia Impact Questionnaire, an unendorsed fibromyalgia-specific tool, and the non-endorsed Visual Analog Scale was registered nearly twice as often as the consensus-body-endorsed Numeric Rating Scale (348 vs. 192 trials).

**Conclusion:**

Fibromyalgia trials have largely resolved whether to measure patient-reported outcomes, but instrument selection remains fragmented despite two decades of guidance, limiting comparability across trials. Requiring a consensus-body-endorsed instrument alongside a disease-specific measure would close this gap without sacrificing disease-specific detail.

## Introduction

Fibromyalgia affects millions of individuals worldwide, yet it remains a poorly understood chronic pain disorder that places a substantial burden on patients through functional impairment and a subsequent reduction in independent living.[1–3] With minimal to no abnormalities on laboratory testing, both its diagnosis and response to treatment are almost entirely dependent on patient report, making patient-reported outcomes (PROs) the primary means of evaluating treatment benefit.[4,5] In recognition of this, the US Food and Drug Administration (FDA) established in 2006 that endpoints intended to reflect how a patient feels or functions should be measured using a validated PRO instrument.[6]

To standardize outcome measurement in chronic pain trials, the Initiative on Methods, Measurement, and Pain Assessment in Clinical Trials (IMMPACT) recommends a core set of patient-centered domains (pain, physical functioning, emotional functioning, and global improvement) with validated instruments for each.[7,8] Because IMMPACT spans chronic pain rather than any single disease, it offers one common benchmark for the many treatments tested in fibromyalgia.[9] Fibromyalgia trials vary widely in the instruments they adopt, often reducing a multifaceted condition to a single-item pain scale.[10] Such variability limits cross-study comparisons, complicating evidence synthesis and clinical decision-making.[11]

ClinicalTrials.gov provides an opportunity to evaluate outcome measurement as planned before trial initiation across a broad cohort, including trials that were terminated, withdrawn, or never reported.[12] However, registrations are often imprecise; many primary outcomes are described too vaguely to identify the intended objectives.[13]

We conducted a cross-sectional analysis of interventional fibromyalgia trials registered on ClinicalTrials.gov. The registration record offers a prospective, largely unexamined window onto how fibromyalgia trials plan patient-reported measurement at registration. The aim of this study was to assess the use of named PRO measures, the proportion of trials that specified a validated instrument rather than a vague or absent outcome, and how closely their instruments aligned with IMMPACT recommendations following their 2005 publication.

## Methods

This registry-based, cross-sectional meta-research study examined how interventional fibromyalgia trials specified PROs at registration using publicly available ClinicalTrials.gov records. The OSU-CHS Institutional Review Board granted the study exempt status (#2026032). The protocol was preregistered on the Open Science Framework (OSF).[14] The full reproducibility package is deposited on OSF and contains the extracted dataset, the frozen instrument dictionary, all derived output files, and the analysis code covering extraction, screening, reconciliation, detection, and figure generation. The analysis code is available on GitHub at fibromyalgia, commit 6fbd7e6, release tag v1.0-verified.[15]

### Search Strategy

Trials were identified by searching the ClinicalTrials.gov web interface for “fibromyalgia” on 14 July 2026, limited to interventional studies with no additional filters. Two reviewers (MM, HK), blinded to each other’s decisions, independently screened the registered condition field, reviewing the brief title when the condition field named no recognized fibromyalgia variant. Because the web interface expands search terms beyond exact matches, the export included both fibromyalgia condition variants and unrelated studies. Eligible variants were recorded to build the programmatic condition-field query; eligible trials not retrievable by condition term were retrieved by NCT number. Excluded trials’ titles informed a title-based exclusion screen.

Agreement between reviewers was assessed using Cohen’s κ (95% CI) and the prevalence-adjusted bias-adjusted kappa (PABAK), with disagreements resolved by consensus.[16] This screening validation reflects eligibility assessment and is distinct from the reviewer–algorithm detection validation reported below for outcome classification. The final reconciled cohort served as the reference standard for evaluating programmatic retrieval.

### Eligibility

Trials were required to be of interventional study type. No restriction was placed on recruitment or study status, trial phase, or country of registration. Trials were excluded only if they did not concern fibromyalgia, including regional pain conditions with distinct outcome frameworks (e.g., myofascial pain syndrome and temporomandibular disorders), using the title-based exclusion screen described above.

### Records from the search strategy

The cohort was then retrieved programmatically from the ClinicalTrials.gov API (version 2), so the cohort can be regenerated from the archived query rather than a static export.[17] Unlike the web interface, which broadens a search term to related condition entries, the API returns only terms named explicitly, so the programmatic query lists every eligible condition variant recorded during screening. Retrieval combined this condition-field query, restricted to interventional studies, with a direct NCT-identifier lookup for eligible trials whose condition field matched no variant. Study type was re-verified, and the title-based exclusion screen was reapplied.

### Data Extraction

The programmatic extraction drew on the records identified through the search strategy above, pulling all data from the ClinicalTrials.gov registry via API version 2 on 14 July 2026. Because the registry updates continuously, all counts and outcome text reflect the records as of that date.

For each trial, we recorded the NCT identifier, title, first-posted date and overall recruitment status, lead-sponsor name and class, registered intervention names and types, and every primary and secondary outcome-measure entry from the API modules. For each outcome, the measure name and description fields were concatenated before detection, because the instrument is at times stated only in the description and name-only matching would produce false negatives.

Outcome text was extracted from each record’s current version rather than its originally registered wording to reflect current instrument use. Pagination traversed all available pages, with the number of retrieved records verified against the API-reported total; any mismatch halted extraction. Duplicate NCT numbers were removed. Extraction was performed in Python 3.13.9.

### Cohort Verification

Before analysis, two checks had to pass. First, the retrieved cohort had to reach at least 90% of the size expected from screening; a shortfall would signal that retrieval had not reproduced the screened cohort and would halt the run. Second, the retrieved cohort had to match the hand-vetted list with at least 90% overlap. Retrieved trials absent from the vetted list were kept, since the vetted list checks retrieval accuracy rather than defining the cohort; vetted trials not retrieved were logged. All analyses used the retrieved cohort.

### Instrument Dictionary

PRO detection ran solely against a frozen, fibromyalgia-specific instrument dictionary. Each entry records an instrument’s canonical name, the text patterns identifying it in outcome text (full name, abbreviations, and spelling variants), and flags for whether it is patient-reported and IMMPACT-endorsed.

The dictionary was built from a discovery pass conducted before extraction of the analytic cohort which queried fibromyalgia trials without the interventional restriction, so candidates reflected the full registry rather than the analytic cohort alone. Candidates were ranked by trial frequency; reviewers merged spelling and acronym variants, excluded non-instruments, and assigned detection patterns anchored to word boundaries. Instruments appearing in at least three trials were retained, with the threshold waived for IMMPACT-endorsed measures.

Before freezing, all patterns were re-tested against the discovery corpus; patterns matching no trials or missing intended name forms were corrected or confirmed as intentional. The candidate tally and frozen dictionary are archived in Online Resource 1.

### PRO Classification

Classification was manual. A named PRO was a validated patient-reported instrument identifiable by name in the outcome text and formed the numerator for completeness and endorsed-instrument analyses. Each trial was assigned hierarchically, on its complete registered outcome text, to one of three mutually exclusive categories: named PRO (at least one dictionary instrument), vague-only (patient-reported constructs described without a named instrument), and no patient-reported measure (outcomes confined to clinician-assessed, laboratory, or physiological endpoints).

A trial was classified as registering an IMMPACT-endorsed instrument if at least one named PRO belonged to the endorsed set, including the Numeric Rating Scale (NRS) for pain intensity; the Brief Pain Inventory (BPI) or Multidimensional Pain Inventory (MPI) for physical-function interference; the Beck Depression Inventory (BDI) or Profile of Mood States (POMS) for emotional functioning; and the Patient Global Impression of Change (PGIC) for global improvement. Because IMMPACT recommends the NRS as its core pain-intensity instrument and the Verbal Rating Scale only as an alternative when numerical ratings are unsuitable, the latter was not counted as endorsed.

### Alignment and Core Outcome Set

Alignment analyses were descriptive and exploratory, intended to characterize how closely registration practice tracked IMMPACT recommendations rather than assess adherence against a predefined threshold. Overall alignment was calculated as the proportion of named PRO trials registering at least one IMMPACT-endorsed instrument in any primary or secondary outcome.

### Detection Validation

Detection accuracy was evaluated independently of screening. A reproducible random sample of 100 trials was drawn from the analytic cohort using a fixed random seed. Two reviewers, blinded to the algorithm’s classifications and working independently, reviewed each trial’s registered outcome text and manually identified all PRO instruments using the frozen dictionary. Reviewers also recorded any apparent PRO instrument absent from the dictionary, allowing omissions from the closed vocabulary to be quantified.

Agreement between reviewers was summarized using percent agreement and Cohen’s κ. Each reviewer’s classifications were then compared with the algorithm’s output to estimate percent agreement and the false-negative rate.

### Temporal Analysis

Fibromyalgia trial registrations were analyzed across their full history without date restriction, indexed by first-posted year. The FDA’s 2006 guidance on PRO measures and the 2005 IMMPACT instrument recommendations anchor the descriptive figures. The latter defines the two epochs used in the alignment comparison: trials posted before 2005 formed the pre-endorsement epoch and those posted after formed the post-endorsement epoch. Endorsed-instrument use was tracked by registration year and compared across epochs, and the composition of three PRO measurement categories was tabulated by first-posted year and plotted as annual proportions. Single-item pain scales were tabulated separately by year despite falling within the named PRO category. Annual registration volume was plotted alongside these proportions; no interrupted time-series model was fitted.

### Statistical Analysis

Analyses were run in Python 3.13.9 (pandas and matplotlib). Completeness was the share of all trials registering at least one named PRO. Endorsed-instrument use was calculated among named PRO trials, reported overall, and IMMPACT domain. Instrument heterogeneity was the count of distinct named PRO instruments detected across the cohort, with per-instrument trial counts ranked by frequency. Concentration was summarized as the share of all PRO instrument occurrences attributable to the most frequently registered instruments. The number of instruments appearing in fewer than five trials is displayed as a cumulative curve. Each instrument was counted once per trial regardless of how many times it appeared in that trial’s outcomes.

Annual completeness trends were tested with linear regression against registration year, one point per year, reporting R^2^, the slope in percentage points per year, and a two-tailed P value. The regression ran twice, across all years with at least one registration and restricted to years with five or more, because sparse years can swing annual proportions; a trend is claimed only if it holds under the stricter fit.

Endorsed-instrument use before and after 2005 was compared with Pearson’s χ^2^ wherever expected cell counts hit at least five in each epoch, and with Fisher’s exact test otherwise. All proportions carry Wilson 95% confidence intervals. Descriptive percentages covering cohort makeup and trial characteristics are reported as point estimates only. α was set at 0.05.

### Reproducibility

Two analysts independently verified the computational pipeline. One analyst developed the pipeline and maintained an ongoing decision log; the second analyst, blinded to that log, independently reran the entire pipeline on a separate machine. Cohort size, completeness, and endorsed-instrument rates were required to match before results were finalized. Record counts were logged at every processing stage, and the verified pipeline was archived on OSF.

### AI Disclosure Statement

We used Claude Code to write and refine the Python extraction, screening, and instrument-detection scripts, to construct the frozen instrument dictionary, and to improve manuscript readability. All decisions regarding study design, methodology, analysis, and interpretation were made by the authors and logged in a version-controlled decisions.md file in the project repository.

## Results

### Cohort and reconciliation

For screening validation, the two reviewers screened 1,151 records returned by the ClinicalTrials.gov web interface, agreeing on 99.3% of them with almost-perfect agreement (Landis-Koch) on the binary include/exclude decision (Cohen’s κ = 0.979, 95% CI 0.963–0.992; PABAK = 0.986). Title-based exclusion left a hand-vetted list of 913 eligible identifiers, and disagreements were resolved by consensus.

Programmatic retrieval returned 1,154 records, which was reconciled against the hand-vetted list of 913 identifiers and matched at 913 of 916 (99.7%). Every vetted trial was retrieved. Three trials were retrieved but did not appear on the vetted list; all three were first posted in 2026, after the list was compiled. These were retained, and the final cohort was 916 trials.

During detection validation, two reviewers independently classified 100 sampled trials into one of three categories: named PRO, vague only, or no PRO. They agreed on 93.0% of trials (Cohen’s κ = 0.745); seven discordant records were resolved by consensus. Because the algorithm distinguishes only whether a trial names a PRO, the reviewer labels were collapsed to that binary for the algorithm comparison, consistent with the pipeline’s handling of nonspecific text as no detected instrument. On this binary classification, the algorithm agreed with the two reviewers on 98.0% and 97.0% of trials. The false-negative rate, defined as trials a reviewer classified as registering a named PRO that the algorithm did not detect, was 1.0% (1 of 100). Reviewers flagged no instruments that appeared to be PRO measures but were absent from the frozen dictionary.

### Trial characteristics

All 916 trials were interventional. At extraction, 577 (63.0%) were completed and 58 (6.3%) terminated, withdrawn, or suspended; the rest were ongoing or of unknown status. Interventions were most often drugs (241, 26.3%) or behavioral (219, 23.9%), and trials could register more than one type. The cohort came from 486 lead sponsors, most frequently Viatris and the Universidad de Granada (16 each).

### PRO measurement categories

**Figure 1:**
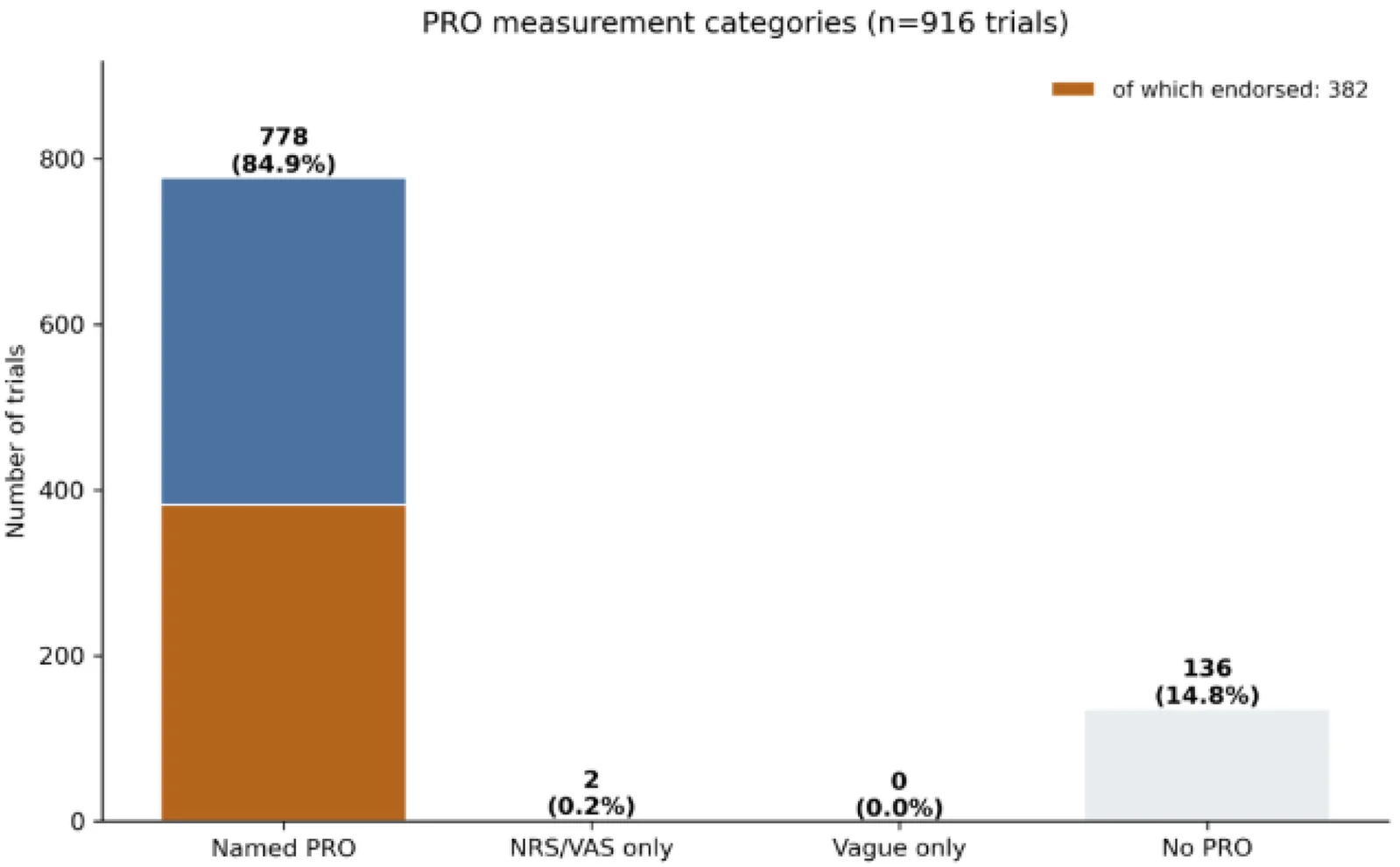
PRO measurement categories across the 916-trial cohort. Bars show trials registering at least one named PRO instrument, only a clinician-administered measure (n=2), or no PRO detected. The shaded portion of the named-PRO bar marks the 382 trials registering an IMMPACT-endorsed instrument. Single-item rating scales (e.g., NRS) count as named PRO instruments

Of 916 trials, 778 (84.9%) registered at least one named PRO instrument. The remaining 136 (14.8%) registered no measure the dictionary detects. Figure 1 shows the category split.

Single-item rating scales counted as named PRO instruments: IMMPACT names the NRS as a core outcome measure, so a trial registering an NRS and nothing else registers a named PRO. VAS appeared in 498 of 916 trials (54.4%), and 69 of the 778 named PRO trials (8.9%) registered a scale and no other named PRO. Trials whose outcome fields carried only non-specific language were not classified as a separate category, so they sit within the 136 rather than in a category of their own.

### Instrument diversity and concentration

**Figure 2:**
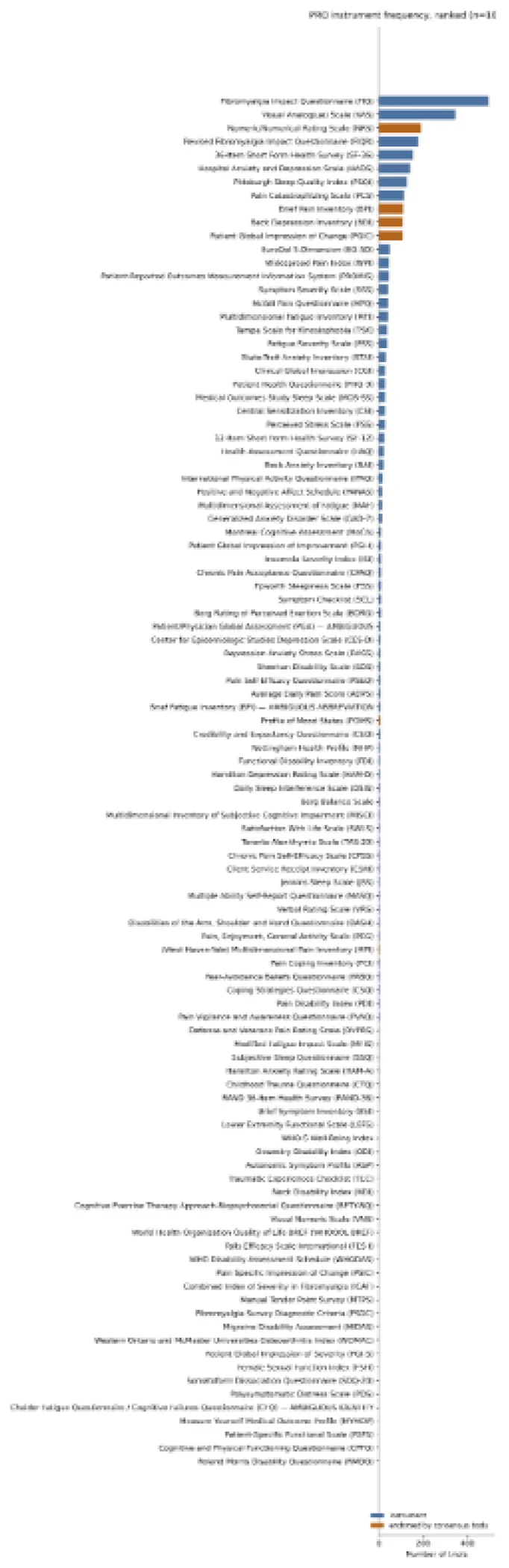
Fibromyalgia measure frequency, ranked by number of registering trials. The 102 named PRO instruments and six clinician-administered measures are shaded separately; the six IMMPACT-endorsed instruments are shaded again within that set. One ambiguous entry (PGA, patient- or physician-reported depending on trial) was excluded from detection and is not shown (10 trials, each counted through another named instrument)

**Figure 3:**
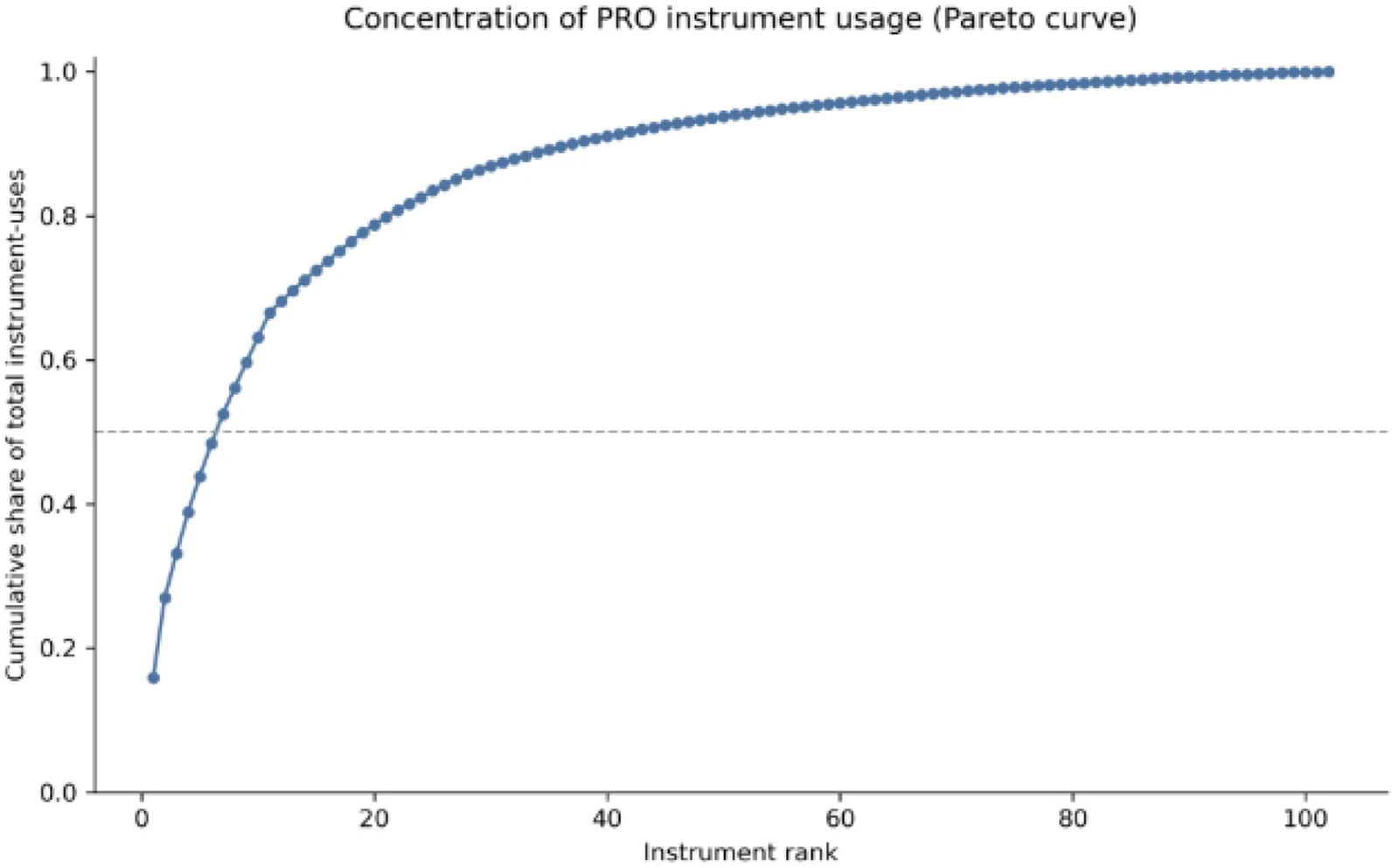
Concentration of PRO measure use. Cumulative share of occurrences (n=3,054, 102 instruments) plotted against measure rank; the dashed line marks 50%. Clinician-administered measures are excluded

The cohort contained 102 named PRO instruments, in 3,054 trial-instrument occurrences. The Fibromyalgia Impact Questionnaire (FIQ) led with 497 trials, followed by the VAS (348), NRS (192), revised Fibromyalgia Impact Questionnaire (FIQR) (179), SF-36 (155), HADS (144), Pittsburgh Sleep Quality Index (128), and Pain Catastrophizing Scale (114). Figure 2 shows the full ranking and the six clinician-administered measures. Two instruments account for 845 of 3,054 occurrences (27.7%), and three account for 1,037 of 3,054 (34.0%). At the other end, 31 of 102 instruments appeared in fewer than five trials, and 3 of 102 in a single trial. Figure 3 plots cumulative share of occurrences against instrument rank. Of the 778 trials registering a named PRO, 136 (17.5%) registered exactly one instrument; the median was three and the maximum sixteen.

### PRO categories over time

**Figure 4:**
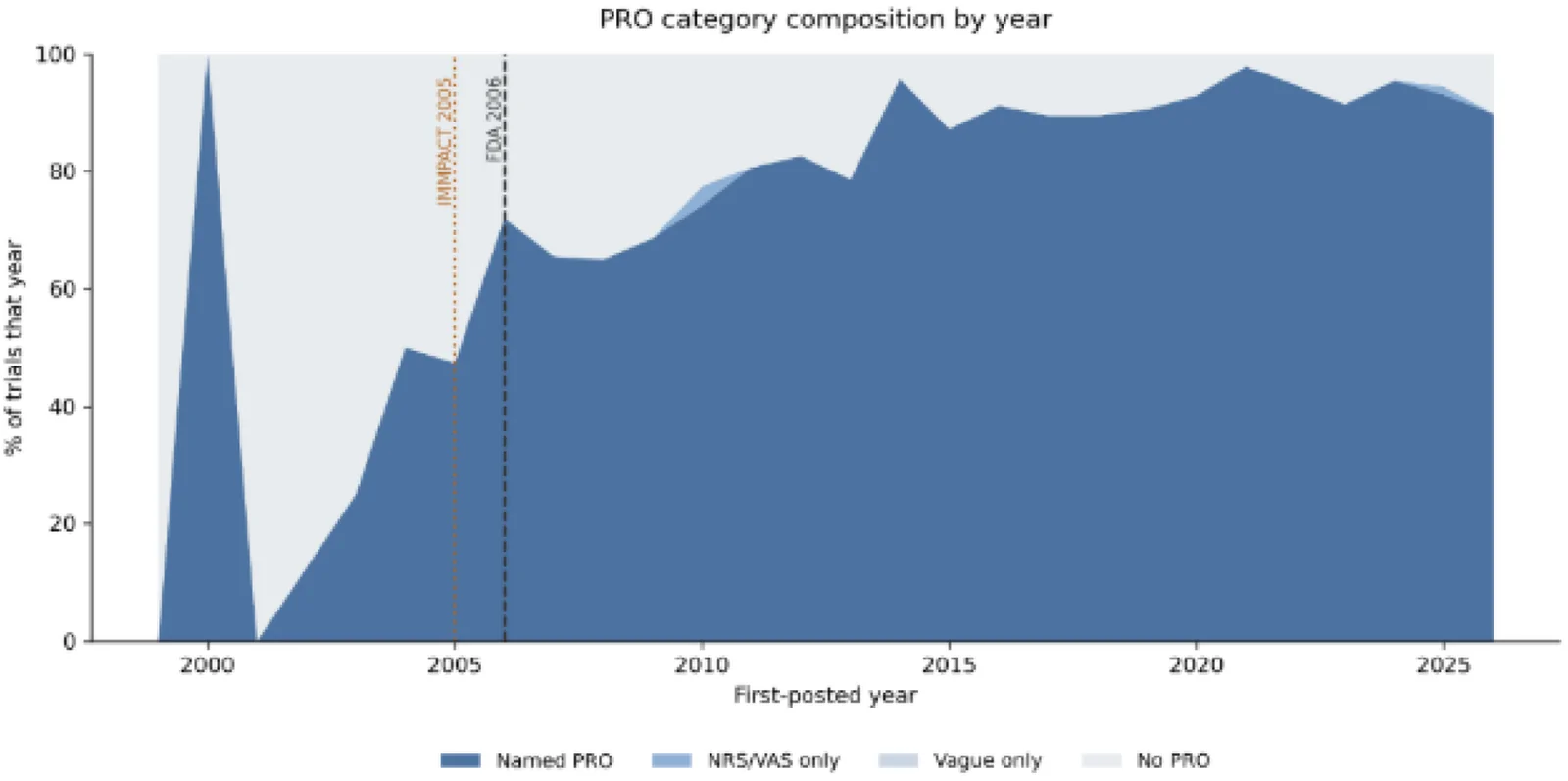
PRO category composition by first-posted year, 1999-2026. Bands show each year’s trials split into named-PRO, clinician-administered-only, and no-PRO-detected categories, summing to 100% (annual n in Fig. S1). Dashed and dotted lines mark the 2006 FDA guidance and 2005 IMMPACT recommendations as reference points only, not analytic cut-offs. 2002 held no registrations; 2026 is a partial year

Registrations run from 1999 to 2026, excluding 2002, which had none. Annual counts range from 1 to 71 (Online Resource 2); four years hold fewer than five trials each (1999, 2000, 2001, and 2003), and the whole pre-2005 period holds 18 trials. Volume sits late in the window: 356 of 916 trials (38.9%) were first posted from 2021 on. The 59 trials recorded for 2026 reflect a partial year with extraction completed on 14 July 2026. Figure 4 shows category composition by year. The named PRO share is 0% in 1999 and 2001 and 100% in 2000, years holding four, three, and one trials. From 2005 it ran from 47.4% to 97.9%, and across 2021 to 2026 it stayed between 89.8% and 97.9%.

### Alignment with the IMMPACT-endorsed PRO instruments

Of 916 trials, 382 (41.7%) were aligned, meaning a primary or secondary outcome field registered an IMMPACT-endorsed instrument, accounting for 382 of the 778 (49.1%) trials with a named PRO. The endorsed set included NRS (192 trials), BPI (110), BDI (108), PGIC (108), POMS (8), and MPI (5).

**Figure 5:**
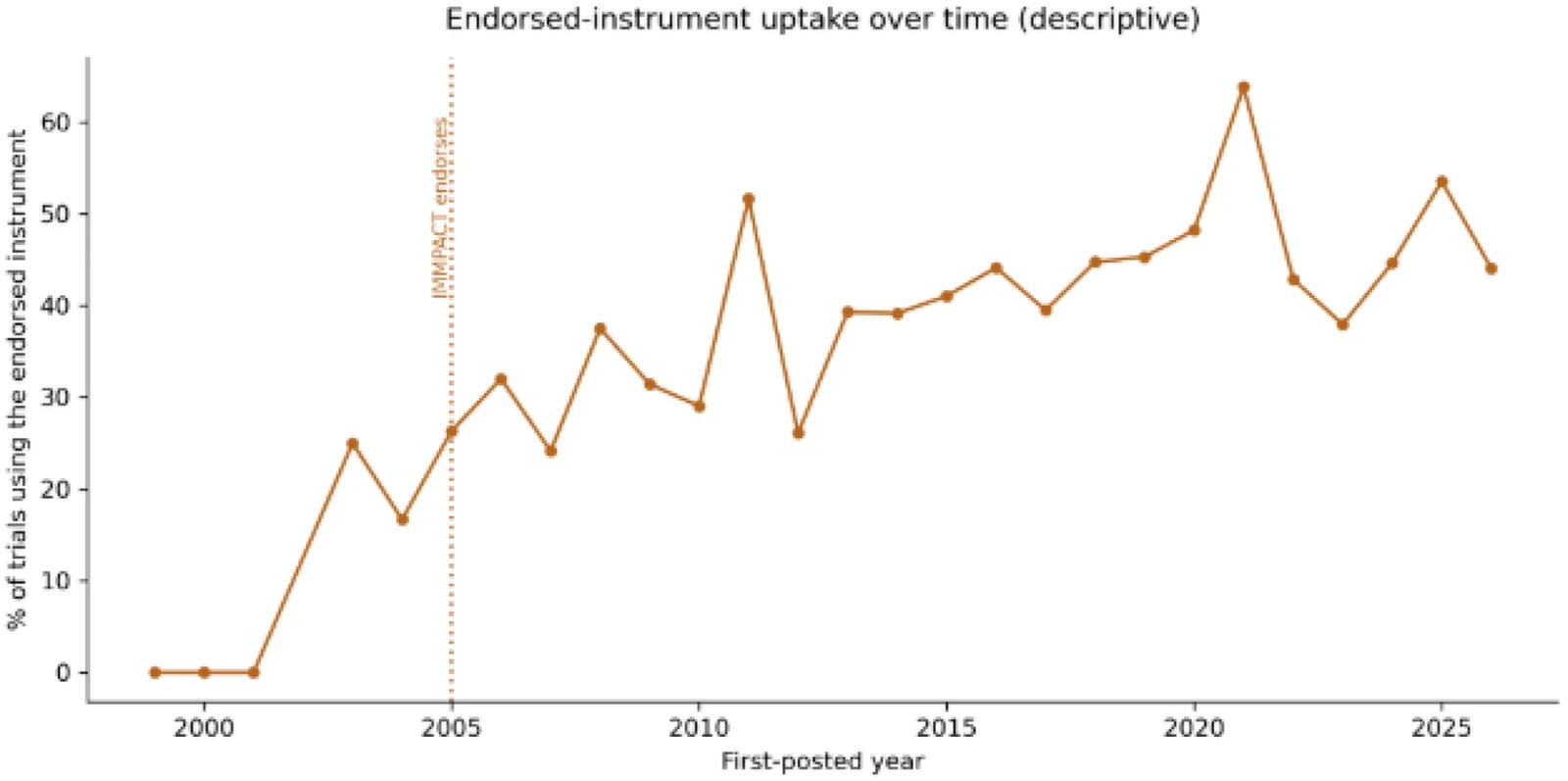
Endorsed-instrument use by first-posted year. Points show the percentage of all trials registered that year, not only named-PRO trials, carrying at least one IMMPACT-endorsed instrument. The dotted line marks the 2005 IMMPACT recommendations

The two most-registered rating scales fall on opposite sides of the endorsed set. The NRS is endorsed and appeared in 192 trials; the VAS is not endorsed, appearing in 348 as the second most registered instrument. Of the 69 trials registering a scale and no other named PRO, 30 were aligned, in every case because the registered scale was the NRS. Figure 5 shows endorsed-instrument use by year.

### Endorsed-instrument use by epoch

Table 1 shows alignment among named PRO trials before and after the 2005 IMMPACT recommendations. Epochs are assigned by registration year, so trials registered at any point in 2005 fall in the post-anchor epoch. Among named PRO trials in the pre-anchor epoch (1999–2004), 2 of 5 (40.0%) were aligned. In the post-anchor epoch (2005–2026), 380 of 773 were (49.2%).

The pre-anchor epoch holds five named PRO trials because the cohort itself holds only 18 trials registered before 2005. The comparison carries almost no information: the smallest expected cell count was 2.5, so Fisher’s exact test was used, and the result was p = 1.000. The confidence interval on the pre-anchor proportion spans 65 percentage points. The comparison is unadjusted.

### Temporal trend testing

Annual named PRO completeness regressed on registration year gives a slope of + 2.51 percentage points per year (R^2^ = 0.540, two-tailed p < 0.001). The fit runs across all 27 years carrying at least one registration and includes the partial 2026 year. Four of those years hold fewer than five trials, and one of them, 2000, holds a single trial that registered a named PRO and so plots at 100%. Restricted to the 23 years holding five or more trials, the slope is + 1.84 points per year (R^2^ = 0.748, p < 0.001). The direction and significance of the trend hold under both fits. The two trials registering only a clinician-administered measure do not support a trend test.

Both rating scales were registered more often across the window (Online Resource 3). The NRS, which IMMPACT endorses, rose by + 1.24 percentage points per year (R^2^ = 0.750, p < 0.001), and the VAS, which it does not, by + 1.72 (R^2^ = 0.674, p < 0.001). Both fits hold when restricted to the 23 years holding five or more trials (+ 1.21, R^2^ = 0.631, p < 0.001 and + 1.20, R^2^ = 0.457, p < 0.001). The VAS was registered more often than the NRS in every year from 2004 onward except 2016, where the two were equal, and 2021, where the NRS was higher. Forty-three trials registered both.

## Discussion

The study examined which PRO instruments interventional fibromyalgia trials registered on ClinicalTrials.gov, how widely that selection is dispersed, and how far it converges on the instruments endorsed by IMMPACT. Across 916 trials registered between 1999 and 2026, most named at least one PRO instrument, yet those trials drew on 102 different instruments, and only about half named one that is endorsed by IMMPACT. Domain-level consensus for chronic pain already exists. IMMPACT names four core domains (pain intensity, physical functioning, emotional functioning, and global improvement) and fibromyalgia additionally carries a disease-specific core domain set agreed upon through patient and clinician input.[7,8,18–20] What the field has not agreed on is which questionnaire answers each specific domain.

The principal finding is not that many instruments were identified but that measurement has remained fragmented despite two decades of standardization efforts. A few dominate, yet investigators keep drawing on alternatives, limiting comparability and synthesis. Because treatment response in fibromyalgia is assessed primarily through PROs, inconsistent measurement complicates the separation of effectiveness from differences in measurement.

The FIQ was the most frequently registered PRO instrument, with its revised version (FIQR) also among the most used.[21,22] Its continued prominence likely reflects its disease specificity, broad domain coverage, and responsiveness to clinical change.[23] Where IMMPACT’s four domains require several separate instruments, a single FIQR covers comparable ground, which may explain why trialists use it. However, widespread adoption of the FIQ has not translated into broader, standardized outcome measurement. The non-endorsed VAS remained substantially more common than the IMMPACT-endorsed NRS despite comparable measurement properties for pain intensity.[24] Although registration of named PRO instruments increased markedly across the study period, IMMPACT-endorsed instruments showed little uptake, with fewer than half of named PRO trials aligned with those instruments more than two decades after endorsement.

One possible explanation for this persistent instrument-level heterogeneity is discordance of outcome assessment between consensus initiatives. IMMPACT provided explicit recommendations for selected instruments, whereas Outcome Measures in Rheumatology Clinical Trials - Fibromyalgia (OMERACT-FM) established the core outcome domains for fibromyalgia without instrument endorsement.[25] Together, these frameworks may have brought attention to PROs without producing convergence on a common set of instruments. Because the divergence lies in instrument choice rather than domain selection, existing trial registration and reporting mechanisms can feasibly address it.

### Implications

This variation is not new; a prior systematic review found that trials rarely assessed all OMERACT-recommended domains and drew on a wide range of instruments to do so.[10] Our approach places the origin of that heterogeneity upstream at registration, before publication selection or reporting choices could shape it. The gap is not unique to fibromyalgia; a survey of acupuncture trials for chronic pain found only a minority used IMMPACT-recommended tools even for the pain domain, suggesting the shortfall sits with IMMPACT uptake generally rather than with fibromyalgia specifically.[26]

Although PRO registration has become commonplace in fibromyalgia trials, instrument selection remains inconsistent. Journals and funders could encourage use of IMMPACT-endorsed instruments alongside disease-specific measures, rather than relying on any named PRO alone. ClinicalTrials.gov outcome fields could adopt a structured instrument vocabulary, surfacing inconsistencies at registration rather than through retrospective analyses. Finally, future consensus efforts could integrate the complementary strengths of IMMPACT and OMERACT-FM. A standardized measurement set pairing a disease-specific instrument with endorsed instruments for each IMMPACT domain could represent one practical approach to satisfying both frameworks while improving comparability across trials.

### Strengths and limitations

This study’s strengths include its registry-based scope, which captures completed, terminated, and unpublished trials that a published-literature review cannot. The cohort was independently reconciled against a hand-vetted trial list at near-total concordance, and screening decisions carried almost-perfect inter-rater agreement. Automated detection was independently validated in a random sample of trial registrations, strengthening confidence in the classification process. In addition, the dictionary and extraction pipeline were finalized before analysis and archived, supporting complete reproducibility of the study workflow.

Several limitations bear on interpretation. Detection is text-based on registered outcome fields, capturing what trials planned to measure rather than what they administered; idiosyncratically named instruments may be undercounted. Alignment was checked against IMMPACT’s instruments, not fibromyalgia’s own OMERACT-FM domains, so a trial using a defensible disease-specific tool such as FIQ scores the same here as a poorly measured one. The pre-2005 comparison rests on very few trials and was not tested statistically. None of the comparisons account for treatment type or trial phase, either of which could carry part of the pattern.

### Future directions

The same approach could be applied to conditions that pair general pain standards with disease-specific instruments. Chronic low back pain and osteoarthritis are candidates, since both already have research on outcome measure adherence.[27,28] A separate step would be mapping FIQ and FIQR items against IMMPACT’s four domains, quantifying how far these instruments cover the recommended domains.

## Conclusion

Most interventional fibromyalgia trials registered on ClinicalTrials.gov now name a PRO instrument, and roughly half of those name one IMMPACT endorses. The field has largely resolved whether to measure PROs, but not which instrument should do so. Selection stays dispersed across more than a hundred instruments, and the two measures trials reach for most, the disease-specific FIQ and the cross-disease VAS, both sit outside the IMMPACT-endorsed set. This locates fibromyalgia’s outcome heterogeneity at the instrument layer, downstream of a domain-level consensus that already exists. Requiring an endorsed instrument alongside a disease-specific measure, and reconciling IMMPACT and OMERACT-FM guidance rather than treating them as separate standards, would close this gap without discarding the disease-specific detail that instruments such as the FIQ provide.

## Supplementary Material

This is a list of supplementary files associated with this preprint. Click to download.

• ESM1.docx

• sUPP.docx

## Figures and Tables

**Table 1.** Alignment among named-PRO trials, by IMMPACT epoch (anchor: 2005).

Epoch	Named-PRO trials	Aligned trials	Alignment % (95% CI)
Pre-anchor (1999-2004)	5	2	40.0 (11.8-76.9)
Post-anchor (2005-2026)	773	380	49.2 (45.6-52.7)
Total	778	382	49.1 (45.6-52.6)
